# Initial development of tools to identify child abuse and neglect in pediatric primary care

**DOI:** 10.1186/s12911-023-02361-7

**Published:** 2023-11-17

**Authors:** Rochelle F. Hanson, Vivienne Zhu, Funlola Are, Hannah Espeleta, Elizabeth Wallis, Paul Heider, Marin Kautz, Leslie Lenert

**Affiliations:** 1 Medical University of South Carolina, Charleston, SC USA; 2 UT Health Houston, Houston, TX USA

**Keywords:** Automated clinical summaries, Child abuse and neglect, Screening, Primary care, Pediatrics

## Abstract

**Background:**

Child abuse and neglect (CAN) is prevalent, associated with long-term adversities, and often undetected. Primary care settings offer a unique opportunity to identify CAN and facilitate referrals, when warranted. Electronic health records (EHR) contain extensive information to support healthcare decisions, yet time constraints preclude most providers from thorough EHR reviews that could indicate CAN. Strategies that summarize EHR data to identify CAN and convey this to providers has potential to mitigate CAN-related sequelae. This study used expert review/consensus and Natural Language Processing (NLP) to develop and test a lexicon to characterize children who have experienced or are at risk for CAN and compared machine learning methods to the lexicon + NLP approach to determine the algorithm’s performance for identifying CAN.

**Methods:**

Study investigators identified 90 CAN terms and invited an interdisciplinary group of child abuse experts for review and validation. We then used NLP to develop pipelines to finalize the CAN lexicon. Data for pipeline development and refinement were drawn from a randomly selected sample of EHR from patients seen at pediatric primary care clinics within a U.S. academic health center. To explore a machine learning approach for CAN identification, we used Support Vector Machine algorithms.

**Results:**

The investigator-generated list of 90 CAN terms were reviewed and validated by 25 invited experts, resulting in a final pool of 133 terms. NLP utilized a randomly selected sample of 14,393 clinical notes from 153 patients to test the lexicon, and .03% of notes were identified as CAN positive. CAN identification varied by clinical note type, with few differences found by provider type (physicians versus nurses, social workers, etc.). An evaluation of the final NLP pipelines indicated 93.8% positive CAN rate for the training set and 71.4% for the test set, with decreased precision attributed primarily to false positives. For the machine learning approach, SVM pipeline performance was 92% for CAN + and 100% for non-CAN, indicating higher sensitivity than specificity.

**Conclusions:**

The NLP algorithm’s development and refinement suggest that innovative tools can identify youth at risk for CAN. The next key step is to refine the NLP algorithm to eventually funnel this information to care providers to guide clinical decision making.

**Supplementary Information:**

The online version contains supplementary material available at 10.1186/s12911-023-02361-7.

## Background

Extensive research over the past several years indicates high rates of abuse, neglect, and other potentially traumatic events for children and adolescents [1–3], with a substantial conferred risk for adverse physical and behavioral health problems [3–5] that often persist in adulthood [6]. According to the most recently available Child Protective Services’ data, 618,000 children were victims of abuse or neglect in 2020, reflecting a rate of 8.4 unique victims per 1,000 youth under the age of 18 [7]. Such high prevalence rates and associated health consequences highlight child abuse and neglect (CAN) as a critical and costly public health concern [8]. However, many children who are at risk for or have experienced maltreatment are not identified nor are they receiving appropriate evidence-based mental health treatment services [3].

Available data indicate that 94–96% of parents seek services for their children through primary care settings as compared to directly accessing mental health treatment providers (i.e., 4–33%) [9]. Annually, as noted by the Centers for Disease Control’s National Health Interview survey, most children are seen in pediatric primary care settings, with 93% of children having a well-child check in 2020 [10]. For many children, a primary care provider may be the only contact with a professional who has the knowledge, expertise, and resources to provide needed assistance. As such, primary care settings offer a unique opportunity to screen for CAN history, risk factors, and related symptomology to increase the likelihood of early identification, further evaluation, and connection to services, when warranted [11–14]. Although previous research supports primary care as a viable setting to conduct screening to identify CAN and CAN risk [11, 15–18], there are inherent challenges in implementing such screenings. Objective, clear evidence of abuse is usually absent, and studies consistently indicate that primary care providers are reluctant to screen for CAN as part of standard practice, [19] with the most frequently cited reasons including time limitations and scope of practice constraints [20–23].

Given the long-term consequences associated with CAN, the 2018 U.S. Preventive Services Task Force on child maltreatment interventions has highlighted a need to prioritize the development and evaluation of effective methods to identify children at risk for maltreatment [24]. With mounting evidence supporting the use of historical patient medical data as a tool to support clinical decision making [25, 26], this could be a valuable resource for early identification and detection of CAN in medical care settings [19, 27]. For example, studies indicate that certain diagnoses and associated clinical symptoms serve as predictors for ongoing or future child maltreatment and that these data could be used to trigger further assessment [27–29]. However, while the use of the electronic health records (EHR) is increasing substantially across clinical care settings, time constraints preclude most primary care providers from being able to review and distill information in EHR that could indicate abuse [16]. Thus, the goal of this research is to develop a strategy to summarize data from the EHR that would identify youth who may be at risk or have experienced CAN. This information could then be conveyed back to primary care providers to guide clinical decision making and potentially mitigate the impact of CAN on child’s mental and physical health.

To achieve this, our plan is to create Automatic Clinical Summaries (ACS) of CAN-related events from data contained within the EHR. ACS technologies extract a structured event, perform natural language processing (NLP), and link different data types [30–35]. By compiling and summarizing data, such as chief complaints, progress notes, radiology reports, consultant notes, outpatient visit notes, and discharge summaries contained within EHRs, ACS have facilitated early detection of several clinical concerns, including physical health problems [36], interpersonal violence [37, 38], postoperative complications [39], and adverse drug reactions [40]. By developing a tool that automatically summarizes evidence for CAN and its impact, we hope to help primary care providers and other clinicians incorporate care for CAN into routine outpatient practice, despite the complexities and difficulties of work in this area. This paper describes the first step in this larger project, with specific aims to 1) develop a CAN lexicon to characterize the population of interest (i.e., children who have experienced and/or are at risk for CAN); 2) develop a database to support CAN term identification in existing EHR, 3) develop and evaluate the newly developed lexicon using NLP tools, and 4) compare more novel machine learning (ML) methods to the lexicon + NLP approach in a pilot study.

## Methods

### Develop the CAN lexicon

#### Characterize transdisciplinary terms likely to be in notes related to CAN

Authors RH, FA and EW generated a preliminary list of commonly used terms to identify CAN, CAN risk factors and related problems. These authors are expert researchers and clinicians (child clinical psychologists and a pediatrician) in CAN identification and treatment, with between 5 to 30 + years of experience in the field. Of note, the authors began with the U.S. federal guidelines that define abuse and neglect at a minimum as *“Any recent act or failure to act on the part of a parent or caretaker, which results in death, serious physical or emotional harm, sexual abuse or exploitation”; or “An act or failure to act which presents an imminent risk of serious harm. “In practice, most US states recognize child abuse and neglect to include physical abuse, sexual abuse, and emotional abuse or neglect* [41].*”* Terms were included based upon shared agreement among these domain experts and were generated for acts of child physical abuse (e.g., hit, kick, punch, shove, use of weapon, etc.), sexual abuse (e.g., rape, molestation, pornography), neglect (e.g., malnutrition; poor living conditions; impaired caregivers due to substance use, mental illness); and emotional abuse (e.g., fear, coercion, etc.). This work was combined with a preliminary keyword search of a text corpus of clinical notes to confirm the presence of concepts and generate new ideas. This initial round generated 90 total terms relevant to CAN for further study.

#### Validate and prioritize CAN terms

The second phase involved validation and prioritization of the terms generated in the initial list. Specifically, we identified an interdisciplinary group of practitioners (i.e., pediatricians, nurse practitioners, pediatric radiologists, and child abuse mental health professionals) from academic medical centers, research universities, and clinical agencies across the United States (e.g., South Carolina, North Carolina, Washington, Pennsylvania, New Jersey, Maryland, Georgia, Oklahoma), who routinely deal with CAN, to review and rate the initial list of terms generated by the study team. An email invitation was sent to *n* = 49 professionals to participate in a brief survey developed for this study (see Supplemental file) as part of a pilot study to generate a list of terms that may be in EHR to suggest a child may have experienced or be at risk for CAN. The email invitation indicated that the goal of the pilot was to identify terms likely to signal CAN, and to this end, professionals were selected because of their expertise in child abuse. The email included a link to a RedCAP survey. In the survey, respondents rated each term’s relevance to CAN, using a 3-point scale: (1 = not important; 2 = somewhat important; 3 = critical for inclusion). Respondents were also provided a free text box to include any additional terms that should be added to a revised list, based on their own interdisciplinary experience and expertise. The survey instructions informed participants that no identifying information was being collected, as the intent was for surveys to be anonymous. Finally, the instructions indicated that a second survey would be sent to the same pool of experts, in approximately two months, to review the free text items endorsed by at least 10% of participants.

After review of the results of the first survey (described below) a second online RedCAP survey that included the (*n* = 59) free text items, generated by at least 10% of Round 1 participants, was sent to the same initial group. Respondents rated these new items using the same Likert scale as above. Items scoring scored at a “2” or higher by at least 80% of participants in either round were included in the final lexicon.

### Develop database to support CAN term identification

#### Source of data

Clinical notes and other text data were drawn from three pediatric primary clinics at an academic health center in the southern United States. Two of the clinics were general pediatric primary care clinics, while the third provides consultative specialty behavioral health care and medication management for children in foster care across the state. The study cohort included male and female pediatric patients, ages 0–19, who visited one of these three pediatric Primary Care Clinics between January 2012-December 2018. Collectively, these clinics served about 6,500 active patients (as measured by the number of patients seen at least once over the past two years) and had a total of 13,000 outpatient visits per year. Most patients were Black/African American (78%) and had insurance coverage through Medicaid (84%).

Patient medical records were obtained from the university’s Enterprise Data Warehouse (EDW), which serves as the data repository for clinical practices of the academic health center. Structured data, including demographics, diagnoses, procedures, and visit tables, were also obtained from the EDW. Records were partially anonymized to enhance patient privacy (a unique research specific identifier was assigned), and the search was confined to the minimum data elements needed to accomplish project tasks. This study was approved by the university’s Institutional Review Board.

### NLP development and evaluation

#### Develop and evaluate NLP and machine learning algorithms to identify CAN from EHR

The goal in this phase was to develop NLP algorithms that could identify notes with CAN-related terms and to highlight concepts within the context of the note for clinician review. CLAMP (Clinical Language Annotation, Modeling, and Processing Toolkit) [42], a comprehensive NLP software designed to analyze EHR data, was used to develop pipelines for CAN-related term identification. CLAMP components have been top-ranked in multiple competitions (e.g., i2b2 NLP challenges) and widely applied to diverse clinical and translational research with over 600 institutional users [43, 44]. Working from the terms endorsed by our experts, we developed an initial customized dictionary for CAN-named entity recognition (NER), customized negations, and Apache UIMA (Unstructured Information Management Architecture) Ruta (Rule-based Text Annotation) for semantic classification of CAN mention(s) in clinical notes.

Selected clinical note types included, but were not limited to, chief complaints, progress notes, radiology reports, consultant notes (i.e., these could include notes from child abuse pediatricians, clinicians on a child protection team, or from any other requested medical consultant), outpatient visit notes, and discharge summaries. The cohort consisted of 20,246 patients seen over the six-year period, with 1,813,186 clinical notes. Duplicated or orphan records were cleaned from the dataset. Clinical notes from a randomly selected subset of patients (*n* = 15,184; 75% of cohort) were reserved as a training set to develop the NLP algorithms; and clinical notes of the remaining 5,062 patients (25% of cohort) were reserved as the test set for the NLP performance evaluation.

Leveraging CLAMP’s built-in default functions modules (i.e., sentence detector, tokenizer, Parts of Speech (POS) tagger, Named Entity Retriever (NER), and assertion identifier), a customized pipeline extracted information relevant to CAN events. We classified CAN-related terms into five subtypes for clinical understanding: ABUSE (e.g., “child abuse,” “violence,” “neglect,” “adverse childhood event,” “childhood trauma,” “alleged”); SEXUAL ABUSE (e.g., “rape,” “SA,” “ inappropriate sexual behavior,” “injury to genital region”); PHYSICAL INDICATION (e.g., “bruise,” “genital/vaginal bleeding”); EMOTIONAL INDICATION (e.g., “threaten,” “humiliate,” “”PTSD”); and SERVICE (e.g., “the name of a local child advocacy center,” “child protective services,” “police”).

Due to a large number of available clinical notes (over 1.8 million), we implemented a strategy sub-setting training set, incrementing the number of clinical notes for training if ideal performance was not achieved. Mining a small random sample of data (14,397 clinical notes from 153 patients), initial Apache UIMA Ruta rules were developed to identify candidate CAN subtypes at sentence level when any of the five subtypes of concepts were identified. CLAMP’s default negation rules (e.g., “deny,” “no”) were applied to exclude negative mentions of CAN terms. Any clinic note with at least one sentence containing an NLP-identified CAN-subtype was classified as a positive CAN note for purposes of record summarization.

#### Evaluate performance of algorithms

The development of NLP pipelines was an iterative process. Two domain experts (authors RFH and FA) manually reviewed the NLP results and labeled false positives for the training dataset. Based on their feedback, a set of customized negations were added to the dictionary to refine the NLP pipelines by further excluding false positives. The CAN dictionary and NLP pipelines were finalized when an ideal performance (precision 90% or above) in the training set was achieved. NLP results from the test set were manually reviewed by the same two domain experts to determine precision. When there was a difference between the two domain experts, it was resolved by review and discussion until common consensus was achieved. A third domain expert was available to assist further review if the discrepancy could not be resolved between the two domain experts. Precision, the proportion of true positives to the total number of NLP-identified cases, was measured for both sentence and document levels.

Finally, we explored a machine learning approach for CAN “topic of sentence” identification, utilizing Support Vector Machine (SVM) algorithms, based on Java libsvm library, with unigrams, bigrams, and tri-grams as features. This was based on the premise that a machine learning approach could help streamline the process of identify patients experiencing or at risk for CAN without relying solely on experts’ manual review of the classification results from the NLP models. Rather than recognizing named entities, the ML approach focused on determining, yes or no, whether a sentence discussed a CAN-related issue. To develop exploratory SVM machine learning NLP approaches, the experts’ manual reviews from the test set (positive and negative CAN sentences) were used as the labeled data for development and evaluation. Two standard measures of sensitivity and specificity at sentence level were reported.

## Results

### Develop CAN lexicon

#### Generate and validate an interdisciplinary list of CAN-related terms for search in health records

As described above, three of the authors generated a preliminary list of 90 commonly used terms to identify CAN, CAN risk factors, and related problems. To validate and prioritize this initial pool of CAN concepts, 25 of 49 invited experts completed the CAN RedCAP survey (see Table 1).

**Table 1:** Number of CAN expert survey participants

Professional Discipline	Survey 1 Invite (n)
Nurse Practitioner	7
Pediatric Sexual Abuse Nurse Examiner	3
Social Worker/LPC	9
Pediatrician	10
Psychologist	12
Psychiatrist	2
Pediatric Radiologist	6
**Total**	**49**

No demographic was information collected at Survey 1, but *n* = 25 respondents completed the RedCAP survey. Of the 49 participants originally invited, the two categories that did not participate in Survey 2 were pediatric sexual abuse nurse examiners and psychiatrists

The average score across the initial pool of 90 items was 2.58, indicating that all were seen as *somewhat important* or *critical* for inclusion and were thus retained for the development of the preliminary dataset. The participants suggested an additional 58 terms (i.e., 58 new terms were endorsed by at least 10% of Round 1 respondents) that were included in the second survey. In the second survey, 12 (48%) of the original 25 respondents, 75% of whom had more than 5 years of experience in the CAN field, rated the new items, utilizing the same 3-point Likert Scale. Of the 58 free response items, 15 were rated < 2 and were discarded. The final pool included *n* = 133 terms that were integrated into the NLP application and used to search EHR documents.

### Database development

Over the six-year period included in the record review, 20,246 patients were seen across the three primary care clinics, generating 1,813,186 clinical notes. The average age of patients was 10 (SD = 4.88) years; with slightly more girls than boys, and higher percentages of Black and non-Hispanic patients (see Table 2). The average number of notes per patient was 90 (minimum 1, maximum 3,049). These data formed the database for subsequent NLP development and evaluation.

**Table 2:** Cohort demographics (*n* = *20,246)*

	**Number of Patients**	**Percentage**
**Gender**		
Male	10,383	51.28%
Female	9,863	48.72%
**Ethnicity**		
Not Hispanic or Latino	14,080	69.54%
Hispanic or Latino	5,793	28.61%
Unknown	373	12.81%
**Race**		
African American	10,788	53.28%
Other	6177	30.51%
Caucasian	2911	14.38%
Asian	212	1.05%
American Indiana or Alaska Native	28	0.14%
Native Hawaiian	11	0.05%
Unknown	119	0.59%

### NLP development and evaluation

A sample of 153 patients from the 15,184 patients in the training set were randomly selected for NLP development and evaluation tasks. Among the 14,397 clinical notes from these patients in the training dataset, the NLP pipeline identified only 422 (.03%) notes (with 1,486 sentences) as having CAN information, and the frequency with which CAN information was identified varied considerably by type of clinical note. The number of terms and percentages of CAN for each type of clinical note is listed in Table 3, and as indicated, positive CAN rates varied across note types. The CAN positive rate was low in common note types, such as Progress Notes, Discharge Summaries, and the After Visit Summary (AVS) SNAPSHOT. The most common types of CAN documentation were found in the Treatment Plan (60%) and SW/CM (Social Worker/Case Manager; 51%) notes; however, the overall number of these two types of notes was very small. Across these two note types, only 27 notes had an NLP identified CAN event). In comparison, other note types contributed a larger portion (*n* = 422–429) of NLP-identified CAN.

**Table 3:** Distribution of CAN positive terms by note type in training set

Note Type	Total Number of Notes	Number of Notes with CAN Positive Term	CAN Positive Percentage
Plan of Care	333	2	0.60
Not Specified	1813	12	0.66
Nursing	134	1	0.75
Emergency Department Notes	518	4	0.77
Patient Instructions	1149	11	0.96
ED AVS Snapshot	472	8	1.69
Telephone Encounter	2051	48	2.34
MR AVS Snapshot	1571	42	2.67
Letter	127	4	3.15
Medical Student	60	2	3.33
Discharge Instructions – Other Orders	58	2	3.45
Sticky Note	27	1	3.70
Emergency Department Provider Notes	281	11	3.91
Progress Notes	3859	175	4.53
IP AVS Snapshot	66	3	4.55
Discharge Summaries	78	8	10.26
H&P	67	7	10.45
Lactation Note	19	2	10.53
Consults	45	8	17.78
Consult NW	11	2	18.18
Social Worker/Case Management	51	26	50.98
Treatment Plan	5	3	60.00

14,397 notes from 153 patients randomly sampled from the training set

*MR AVS* Medical Record After Visit Summary, *IP AVS* Inpatient After Visit Summary, *H & P* History and Physical examination, *Consult NW* Note Writer

**Fig. 1:**
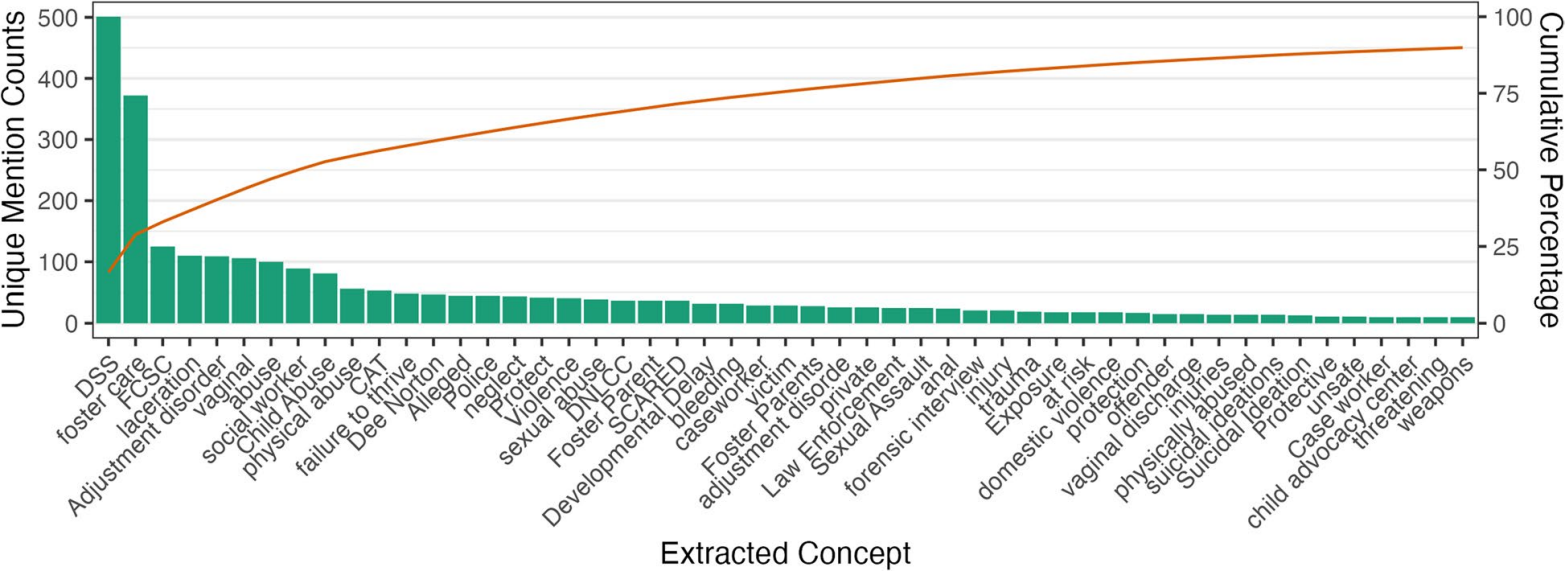
Top CAN entities

The domain expert review confirmed 1,349 sentences with CAN mentions as true CAN cases with mutual agreement. Specifically, there were 134 terms relevant to CAN. The most common terms were child protective services, “foster care,” Foster Care Support Clinic (“FCSC”), “abuse,” “physical abuse,” “Case Manager,” “Child Abuse Pediatrics,” a child advocacy center, “neglect,” and “foster parents” (Fig. 1. *Top CAN Entities*).

**Fig. 2:**
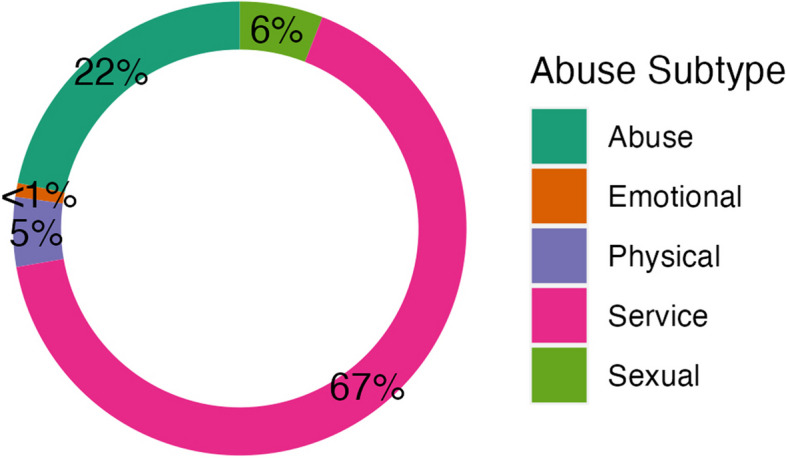
Distribution of CAN hits by subtype in the training set

Figure 2 (*Distribution of CAN Hits by Subtype in the Training Set*) demonstrates the distribution of the 5 subtypes of CAN (i.e., PHYSICAL INDICATOR, EMOTIONAL ABUSE, SEXUAL ABUSE, ABUSE, AND SERVICE) identified in these 1,349 confirmed CAN sentences. SERVICE was the most frequently identified subtype (67%), followed by ABUSE (22%), SEXUAL ABUSE (6%), and PHYSICAL INDICATOR (5%), with no references to EMOTIONAL ABUSE in the training data set.

**Fig. 3:**
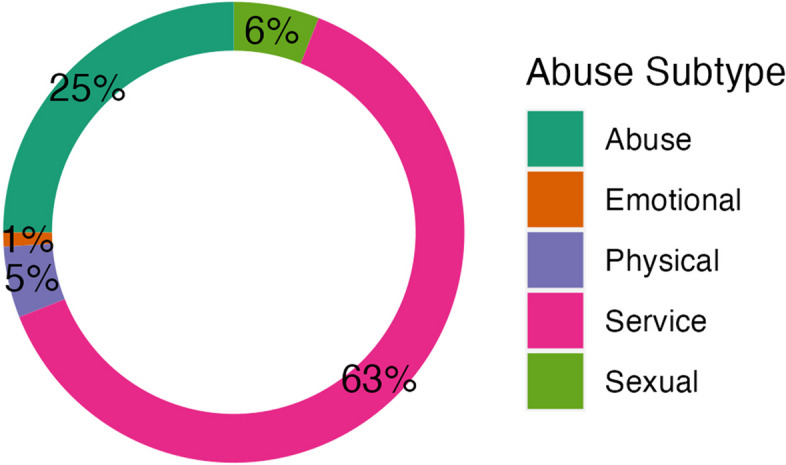
Subtype of CAN positives in the training set: subset of physician notes

**Fig. 4:**
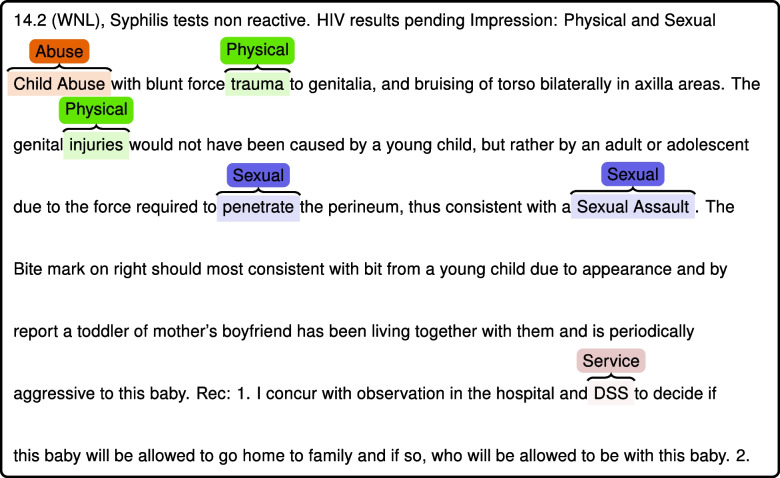
An example of NLP CAN case virtualization CLAMP

The subset of clinical notes (*n* = 875 sentences; Progress Notes, ED notes, ED AVS SNAPSHOT, ED PROVIDER NOTES, Discharge Summaries) from physician providers followed a highly similar pattern (Fig. 3) [(i.e., SERVICE was the most highly identified subtype (63%) followed by ABUSE (25%)]; indicating very few differences between physicians’ notes as compared to those from other providers. In other words, the distribution of subtypes of CAN positives for the physician providers was similar to the full training data set (Fig. 3. *Subtype of CAN Positives in the Training Set: Subset of Physician Notes*). Figure 4 shows an example of the CLAMP virtualization of NLP identified CAN information within clinical notes. It highlights both NLP identified CAN entities and subtypes.

A random sample of 51 patients from the test set, with a total of 4,854 clinical notes, were used to evaluate the performance of final NLP pipelines. The precision was 93.8% for the training set and 71.4% for the test set (Table 4). The decreased precision in the test set can be mainly attributed to false positives introduced by five terms: “case worker,” “case manager,” “incarcerated,” “law enforcement,” and “police”. Although these terms were valid CAN terms during the NLP pipeline development, manual review suggested these terms were not specific enough to identify a CAN incidence, if there was no clear supportive information nearby within the note.

**Table 4:** NLP performance in training and test dataset

Resource	Sample	NLP identified CAN	Manual review identified CAN	Precision
Training Document	14,397	422	396	93.8%
Training Sentence		1,486	1,349	93.8%
Test Document	4,854	105	78	74.2%
Test Sentence		311	222	71.4%

Other major reasons for false positives included 1) the subject of CAN was not a patient (e.g., “Per the grandparents later, mother had one date rape at about age 15, then one sexual assault about 2 years ago.”); 2) the patient was a perpetrator of an abusive incident instead of a CAN victim (e.g., “History of violence: yes—stood over aunt with knife attempting to stab her”); 3) failure to parse complex and agrammatical medical language (e.g., “sexual abuse health since last visit: No illnesses, ER visits, or hospitalizations”); 4) could not clearly distinguish a clinical event from a CAN event (e.g., “First Aid: Burns Call 911 immediately if the victim has any of the following: Symptoms of shock Trouble breathing Second—or third—degree burns over a large area, such as an entire leg or back” or “STD (sexually transmitted disease”); and 5) templated information (e.g., “Provider: N / A Primary Care Physician ( PCP): MUSC Foster Care Support Clinic, Abbreviations: AV = alleged victim, AO = alleged offender”).

#### Machine learning approach identifying sentences with a CAN subject

The dataset for machine learning consisted of 311 NLP identified candidate sentences from the test set for the rule-based approach. Among 311 sentences, 222 (71.4% and representing 17 unique patients) were labeled as CAN subject cases, and 89 (28.6% and representing 15 unique patients) were labeled as non-CAN, by domain-expert manual reviews. The training set included 281 sentences, and the test set had 30 sentences. Both the training and test datasets had the same distribution of CAN (71.4%) and non-CAN cases (28.6%). In the test dataset, SVM algorithm classified 24 CAN and 6 non-CAN cases. Compared with the gold standard (22 CAN cases and 8 non-CAN), SVM correctly identified 22 CAN cases and had 2 false positives, and SVM-identified 6 non-CAN that were all true negatives. The performance of the SVM pipeline was 92% for CAN and 100% for non-CAN. Despite a small test dataset, the SVM algorithm performance was observed to be optimal for both positive and negative cases of CAN.

## Discussion

This study describes our initial work to create a CAN lexicon that can be used in developing a summarization tool to help clinicians rapidly identify cases with potential CAN related events. It is one of the first to utilize expert review and consensus, along with NLP identification, to develop a lexicon to identify CAN related concepts. In general, CAN is an under-reported, under-coded, and under-documented healthcare issue [45]. This work contributes to efforts to improve early identification methods for children experiencing or at risk for CAN, with the hopes that this will enable providers to intervene quickly and potentially mitigate future CAN and associated negative sequalae [46].

One of our initial findings included an interdisciplinary lexicon of terms related to CAN that was tested at a single health care institution. Somewhat surprisingly the CAN lexicon was most frequently documented in the *service* the child received (i.e., child protective services, child advocacy centers), whereas the specific subtypes of abuse (i.e., sexual, physical, emotional) were rarely documented in the EHR. Further, there were highly similar patterns across physician and other interdisciplinary providers, findings which differ from a recently completed qualitative study to inform development of a machine learning-based risk model to identify potential CAN in pediatric emergency departments [27]. Specifically, Landau et al. [27] conducted 20 interviews with a diverse sample of clinicians, working in a single pediatric, tertiary care ED, to learn about their documentation practices for CAN. In the current analytic sample, documentation varied across health disciplines (i.e., physicians, nurses, social workers) in the terms used to describe CAN, the types of notes where CAN was documented, and the styles of documentation. For example, nurses favored brief clinical notes for documenting CAN concerns, whereas physicians provided more detailed reports and used both clinical notes and structured fields within the EHR. These disparate findings could reflect differences in the work context – the ED versus primary care settings – and/or region – northeast versus southeast United States. Notably, both studies focused on a single practice, highlighting the importance of additional work to examine CAN documentation and identification across practice and regional settings.

In addition to the lexicon, we created a large dataset for further research on application of NLP methods for identification of CAN, with a subset (i.e., 1% of data labeled by the manual review) labelled with CAN findings by expert review. Future work will expand this dataset. A limitation at present is that the dataset may contain names or other text data that identifies the patient or family. Future work will anonymize the dataset and make it available publicly. The manually reviewed dataset also has a higher rate of positive examples of CAN than a true ecologically valid dataset would, which we will try to address in expanding the dataset for future work.

The results show that it is possible to search for and find concepts within diverse records related to CAN using “traditional” NLP methods. While performance of the screening was relatively high in the training data sets, it fell short in the test sets. While the 75% precision may be further improved, it may be acceptable when the intent is to target notes for clinical review to determine the significance of a phrase or sentence as part of a focused clinical summarization tool. User testing is necessary to determine the acceptable rate of false positives for CAN concepts in a focused ACS tool. We posit that 100% precision is probably not necessary, given the plans to present these results in the context of a clinical note, which would have been confirmed by the ACS, as shown in Fig. 4 (*An Example of NLP CAN Case Virtualization CLAMP)*.

The specific types of notes with CAN-related terms (i.e., NLP identified positives) revealed the unevenness of documentation of CAN issues in medical records. CAN related terms were rarely seen in primary care notes, even in a population where CAN should be common. In particular, less than 1% of ED notes and only about 4% of primary care notes had mentions of the terms included in our lexicon. This suggests that either the lexicon is missing critical concepts (however, there was no evidence of this on manual review) or that clinicians in these settings may need prompts to encourage documentation of risks and/or effects of CAN in routine care. The “SERVICE” subtype was the most commonly identified across providers (physicians as well as other interdisciplinary providers). Service is a dominate subtype reflecting that a patient who seeks care in these services was usually a victim of CAN. Interestingly, the “PHYSICAL” subtype consisted of very relevant terms, such as *bleed* and *fracture*. However, these terms also indicated clinical findings that may not be caused by CAN and may instead reflect false positives.

Determining whether the overall content of the sentence was relevant to CAN was a somewhat more accurate approach in preliminary testing that could be combined with the lexicon + NLP approach. The SVM algorithm correctly identified 92% of sentences about CAN incidents and 100% of non-CAN incidents. Future work will examine prediction of CAN risk across a patient’s complete record. Estimates of the probability of a CAN related diagnosis may be a useful decision support technique to help clinicians explore risks and evidence for CAN in patient/family member interviews and examinations. This may also improve documentation of risk factors and/or effects in subsequent notes.

### Limitations and future directions

While promising, the current work is not without limitations. First, we elected to collect minimal demographic information from respondents completing the surveys to validate and prioritize CAN terms. While our intent was to maximize likelihood of participation, we recognize that this precluded our ability to ensure variability across clinical roles and geographic distribution. Second, although we collected data from three clinical sites, we utilized a single hospital-system with a predominately African American and lower SES population (i.e., 84% had Medicaid and 53% identified themselves as Black/African American). Future research should incorporate data from additional hospital systems and include hospitals with greater patient racial and financial diversity to ensure generalizability of CAN lexicons and NLP analyses. The larger corpus with more varied patient demographics will allow additional equity and fairness evaluations to help distinguish between algorithmic bias in detecting CAN versus clinical bias in documenting CAN. Named entities related to the delivery of social services for CAN may be a particular problem for NLP analyses. In general, anonymization methods censor named entities, but when these entities are “shorthand” for the providers of social services (as opposed to the names of relatives), filtering out these names would result in loss of critical information.

It is also important to acknowledge that this tool was not designed to identify occult cases of CAN, since we focused our dictionary terms on concepts that domain experts would consider indicative of CAN or risk of CAN. We are currently collaborating with a team that analyzed instances of reported CAN at the note level, which potentially allows for the discovery of occult occurrences. There are benefits to both approaches (that is, the sentence-level approach focused on risk, like we took, and the document-level approach focused on reports, that our colleagues took). Our collaboration is, in part, to study the trade-offs between the approaches and how the different approaches interact with or are sensitive to clinician and algorithmic bias. This design decision was partially why we focused on Precision, rather than Recall, as a target measure.

Finally, alert fatigue is an important factor to consider in development of a clinician decision support (CDS) tool, given the time constraints and competing priorities for providers in clinical settings. We want to highlight that the focus of this manuscript was on development of the NLP tool and that we have not yet created the CDS tool. We are currently conducting qualitative interviews with primary care providers to learn more about the best ways to reduce alert fatigue that will inform our plans to develop a CDS.

## Conclusions

In summary, the NLP algorithms’ development and refinement addressed a lack of innovative tools to harness underutilized medical data for crucial clinical decision-making and practical applications to address CAN, a significant public health concern. The current work demonstrated utility for future efforts to identify CAN through innovative machine learning techniques. Our next steps are to refine the NLP algorithm through additional testing with a larger, more diverse sample. This will inform development of a clinical decision support tool that could be integrated into routine care to provide guidance for providers serving this vulnerable population.

### Supplementary Information

**Additional file 1.** CAN ACS-Survey.

## Data Availability

The datasets used and/or analyzed for the current study are available from the corresponding author upon reasonable request.
