# Supplementary material for: Initial development of tools to identify child abuse and neglect in pediatric primary care
Source: BMC Med Inform Decis Mak. 2023 Nov 17;23:266. doi: 10.1186/s12911-023-02361-7 (PMC10656827; doi:10.1186/s12911-023-02361-7)
Supplement: Supplementary file 1 — Additional file 1. CAN ACS-Survey. [file 12911_2023_2361_MOESM1_ESM.pdf]

CAN ACS-Survey

Please complete the survey below.

Thank you for your willingness to participate in this brief survey as part of our pilot study. Aims of this study are to develop and evaluate the use of a new tool that would guide clinical decision making for primary care providers of children at risk for child abuse and neglect (CAN). As a first step in this process, we must generate a list of CAN-related terms that may be in medical records to be sure we capture as many cases as possible. For this, we are reaching out to a variety of child abuse professionals to help us generate a comprehensive list, using the Delphi technique, which involves two brief online RedCAP surveys (described below). We are inviting you to participate given your expertise in this area.

Below is a preliminary list of terms that are likely to be included in medical records as indicators of child abuse or neglect. We are asking you to review each term and provide a score on its relevance to child abuse or neglect, using a 3-point Likert scale.

		Not important	Somewhat important	Critical for inclusion
1)	Abuse	○	○	○
2)	Abusive relationship	○	○	○
3)	Adjustment Disorder	○	○	○
4)	Alleged/Allegations	○	○	○
5)	Beat (all versions of the word- e.g., beaten, beating)	○	○	○
6)	Belittle (all versions of the word- e.g., belittling, belittled)	○	○	○
7)	Berate (all versions of the word- e.g., berating, berated)	○	○	○
8)	Black eye	○	○	○
9)	Bleed/bleeding/bled/blood (all versions)	○	○	○
10)	Break/Broke (all versions of the word- e.g., broken)	○	○	○
11)	Bruise (all versions of the word- e.g., bruises, bruising)	○	○	○
12)	Bruising	○	○	○
13)	Burned	○	○	○
14)	Case worker	○	○	○
15)	Child abuse	○	○	○
16)	Child Advocacy Center (including any named, such as Dee Norton, Dorchester Children	○	○	○
17)				

Child Protective Services (CPS)	○	○	○
18) Child welfare	○	○	○
19) Choke (all versions of the word- e.g., choking, choked)	○	○	○
20) Cigarette burn	○	○	○
21) Coerce (all versions of the word- e.g., coercion)	○	○	○
22) Concussion	○	○	○
23) CSA	○	○	○
24) Danger (all versions of the word	○	○	○
25) Dirty	○	○	○
26) Domestic violence, DV, domestic abuse	○	○	○
27) DSS (abbreviation and full name, Department of Social Services)	○	○	○
28) Emaciated	○	○	○
29) Emotional abuse	○	○	○
30) Exploit (all versions of the word- e.g., exploited, exploitation)	○	○	○
31) Fall (all versions of the word- e.g., fell)	○	○	○
32) Fear/afraid/scared	○	○	○
33) Fondling	○	○	○
34) Forensic interview	○	○	○
35) Foster care	○	○	○
36) Fractures (all versions of the word- e.g., fractured)	○	○	○
37) Genital bleeding	○	○	○
38) Genital pain	○	○	○
39) Harsh discipline	○	○	○
40) Hemorrhage/Hematoma	○	○	○
41) Hit/Slap/ Smack (all versions - e.g., smacked, smacking)	○	○	○
42) Homeless	○	○	○
43) Hungry	○	○	○
44) Impaired caregivers	○	○	○
45) Inadequate shelter	○	○	○
46) Injured	○	○	○
47) Interpersonal violence, IPV	○	○	○
48)			

	Kick (all versions of the word- e.g., kicked, kicking)	○	○	○
49)	Kinship care	○	○	○
50)	Malnutrition/malnourished	○	○	○
51)	Mandated report	○	○	○
52)	Marks	○	○	○
53)	Molest (all versions of the word- e.g., molestation, molested, molesting)	○	○	○
54)	Neglect	○	○	○
55)	Nonaccidental	○	○	○
56)	Odor	○	○	○
57)	Offender	○	○	○
58)	Perpetrator	○	○	○
59)	Physical abuse	○	○	○
60)	Police	○	○	○
61)	Poor living conditions	○	○	○
62)	Pornography	○	○	○
63)	Post-traumatic stress disorder/PTSD	○	○	○
64)	Private parts	○	○	○
65)	Psychological abuse	○	○	○
66)	Punch (all versions of the word- e.g., punched, punching)	○	○	○
67)	Push (all versions of the word	○	○	○
68)	Rape	○	○	○
69)	Run away (all versions of the phrase- e.g., ran away)	○	○	○
70)	SA	○	○	○
71)	Sexual abuse	○	○	○
72)	Sexual assault	○	○	○
73)	Shake (all versions of the word- e.g., shaking, shook)	○	○	○
74)	Shove (all versions of the word- e.g., shoved, shoving)	○	○	○
75)	Skeletal/osseus survery	○	○	○
76)	Sodomy	○	○	○
77)	Stab (all versions of the word	○	○	○
78)	Substance use/abuse	○	○	○
79)	Suicide (attempted/ideation)	○	○	○
80)				

Trafficking (all versions of the word- e.g., trafficked)	◯	◯	◯
81) Trauma	◯	◯	◯
82) Unkempt	◯	◯	◯
83) Unlawful Conduct to a Child	◯	◯	◯
84) Unsafe	◯	◯	◯
85) Victim	◯	◯	◯
86) Violence	◯	◯	◯
87) Weapon	◯	◯	◯
88) Witnessed violence	◯	◯	◯
